# Longitudinal Outcomes of Neurofeedback and Hyperbaric Oxygen Therapy in Treating a Traumatic Brain Injury Patient: A Case Report

**DOI:** 10.7759/cureus.64918

**Published:** 2024-07-19

**Authors:** Tami Peterson, JeAnnah Rose AbouAssaly, Wendy Bessler, Sheila Burgin, Robert Sherwin, Frederick Strale,

**Affiliations:** 1 Hyperbaric Oxygen Therapy, The Oxford Center, Brighton, USA; 2 Neurofeedback, The Oxford Center, Brighton, USA; 3 Neuropsychiatry, Advanced Neuropsychiatric Specialists, Farmington Hills, USA; 4 Hyperbaric Oxygen Therapy, Wayne State University School of Medicine, Detroit, USA; 5 Biostatistics, The Oxford Center, Brighton, USA

**Keywords:** brain maps, hyperbaric oxygen therapy (hbot), neurofeedback therapy, pre-post treatment, qeeg, quantitative electroencephalogram, severe traumatic brain injury, traumatic brain injury(tbi)

## Abstract

Severe Traumatic Brain Injury (TBI) is a significant health issue, with neurofeedback and Hyperbaric Oxygen Therapy (HBOT) as potentially effective treatments. Neurofeedback uses operant conditioning for real-time psychological and physiological awareness, and HBOT increases blood oxygen levels, potentially enhancing cognitive abilities and the body's innate healing processes and reducing symptoms.

On July 30, 2018, a 33-year-old female runner was hit by a car going 40 mph and thrown 30 feet, resulting in a severe TBI and a seven-week coma. After seven months of intensive rehabilitation, she started HBOT and neurofeedback treatments in November 2021, as recommended by her neuropsychiatrist. These treatments led to noticeable improvements in her cognition, sleep, conversation skills, emotional control, and relationships by January 2022.

By December 2023, after 195 neurofeedback and over 300 HBOT sessions, she reported further improvements in various cognitive and emotional aspects and daily activities like feeding, toileting, grooming, and communication. Post-treatment quantitative electroencephalogram (qEEG) results in June 2024 showed moderate to large effects on her brain’s average frequency band parameters (g = .612) and small to moderate average effects on 19 scalp electrode placement sites outcomes (uV2 g=.339 and Hz g=.333). This indicates significant progress in her recovery journey over a 31-month treatment period.

This patient’s case demonstrated noteworthy improvements in cognitive variables, namely, feeding (p=0.046), toileting (p=0.046), grooming (p=0.046), and communication abilities (p=0.046) per the objective measures, Disability Rating Scale (DRS) and the Glasgow Outcome Scale Extended (GOSE). Based on the qEEG effect sizes, DRS, and GOSE results from the pretest (2021) and posttest (2024), the patient has made noteworthy gains in brain recovery and overall quality of life.

## Introduction

Traumatic brain injury (TBI), which can range from mild to severe, has the highest incidence of all common neurological disorders and poses a substantial public health burden. TBI is increasingly documented not only as an acute condition but also as a chronic disease with long-term consequences, including an increased risk of late-onset neurodegeneration [1]. TBI remains one of the leading causes of death and disability worldwide [2]. The symptoms of an injury can vary based on its severity and location and may encompass cognitive impairment, reduced physical capabilities, emotional and mood disturbances, and sleep disorders. There were approximately 214,110 TBI-related hospitalizations in 2020 and 69,473 TBI-related deaths in 2021. This represents more than 586 TBI-related hospitalizations and 190 TBI-related deaths per day. These estimates do not include the many TBIs that are only treated in the emergency department, primary care, urgent care, or untreated [3]. Even though there has been extensive research on traumatic brain injuries, there is still a demand for additional studies on treatments that are clinically effective for neurological conditions [3,4]. 

Neurofeedback treatment for TBI

Neurofeedback is a technique that utilizes operant conditioning to help patients become more conscious of their physiological responses in real time. With practice and feedback, patients can learn to control these automatic responses more efficiently, reduce symptoms, enhance performance, and boost well-being [5-7]. Different types of physiological measures are used in this process, including electroencephalogram (EEG)-neurofeedback. These measures can be used individually or in combination to provide data to the user, typically through visual and audio displays, under the guidance of a trained clinician. Reinforcements, such as manipulating computer game elements based on physiological changes, help train users to become more aware of their physiological responses to emotional, cognitive, or physical stimuli. The goal is for users to gain more control over these responses and be able to apply this ability in real-life settings [5-7]. 

Neurofeedback has proven effective in treating TBIs and strokes. It has been utilized with success in rehabilitating learning and memory [8, 9], attention [10], and even motor skills [11] in patients with TBIs and strokes. The therapy's impact can be seen in the alterations in neuropsychological scale scores and specific features of the resting EEG [11-13]. 

Chen et al. [14] conducted a randomized controlled trial with 87 patients using their Loreta Z-Score Neurofeedback (LZNFB) and Theta/Beta Neurofeedback protocols. Their LZNFB protocol aimed to improve cognitive function by training individuals to achieve a power activity of 14 to 18 Hz (low-beta) in a 7-voxel cluster of neurons in the anterior cingulate gyrus. Their study calculated 5700 EEG metrics and continually compared them in real-time to a reference database to derive Z-scores. Patients received positive feedback from visual or audio cues in the video when their EEG activity achieved normative values as defined by prespecified criteria. The prespecified criteria included: Z-scores remained within a target window of ±1.5 standard deviations of the normative mean. A percentage of the 5700 Z-scores was manually adjusted to fall within the target window 60% to 70% of the time. A reward rate of 60% to 70% for 24 to 36 rewards per minute was maintained during each session.

With Chen et al.'s Theta/Beta neurofeedback protocol, the electrodes were placed at the Fz and Cz sites with a linked ears reference (A1). The training interfaces of 10 different games were presented on a computer screen. All games displayed the patients’ response scores, synchronized with auditory feedback whenever the task conditions were met to Increase the beta power (13-20 Hz). Inhibit the theta power (4-8 Hz) relative to the baseline values determined at the beginning of a training session [14].

Chen et al. found that the neurofeedback group exhibited significantly more significant improvements in immediate recall, delayed recall, recognition memory, and selective attention compared with the control group; the theta/beta neurofeedback group improved in only immediate memory and selective attention at p < .05 [14]. The total Community Integration Questionnaire-Revised (CIQ-R) scores of the neurofeedback group after treatment were greatly improved compared to those of the control group. Consecutive neurofeedback sessions achieved therapeutic effects in memory, attention, and productive activity, whereas theta/beta neurofeedback improved memory and attention in patients with TBI [14]. 

Gupta et al. reported on 14 patients with TBI in the post-injury period ranging from three months to two years. All participants underwent twenty sessions of neurofeedback training - the training aimed to reduce the theta-alpha amplitude ratio by reinforcing alpha and inhibiting theta activity. The active sites for the electrodes were fixed at O1 and O2 locations per the 10-20 International system [15]. Each reference electrode was placed on the mastoid, and the ground electrode was placed on the forehead. An abrasive gel was used to clean and prepare the scalp/skin, then mounting the electrode using a conductive paste. Before the procedure, the goal and nature of the task were thoroughly explained to the participant. The display screen was selected based on the participants’ choice. Participants were instructed to relax and focus on the screen. Rewards were given through visual feedback (i.e., an increase in the score) displayed on the screen. Each neurofeedback training session lasted for a 40-minute duration [15].

Comparisons were made between the baseline and post-neurofeedback training on post-concussion symptoms and electrophysiological variables. The results showed a significant decrease in the severity of post-concussion symptoms after neurofeedback training [15]. A consistent trend of reduced amplitude ratios of slow and fast waves was also observed after neurofeedback training, although this was not significant across all brain regions. The study suggests that neurofeedback training may play a role in improving post-concussion syndrome and normalizing quantitative electroencephalogram (qEEG) in TBI patients. This has implications for clinical decision-making, suggesting neurofeedback as a potential alternative treatment option for TBI patients [15]. 

Vilou et al. [16] reported results from Munivenkatappa [17] and colleagues who conducted training with two patients aged 15 years old who had moderate TBIs. Computer tomography scans indicated potential diffuse axonal injury. Both patients underwent 20 sessions of neurofeedback, each session lasting 40 minutes, three times a week for two months. The neurofeedback protocol focused on theta (4-7 Hz) and alpha (8-12 Hz) wave frequencies. Assessments before and after the neuropsychological intervention (using the Rivermead concussion symptoms scale and National Institute of Mental Health and Neurosciences (NIMHANS) Neuropsychology Battery revealed enhancements in mental speed, working memory, and visual memory retrieval [16,17]. 

Rostami et al. [18] published a randomized controlled trial involving 13 patients with moderate TBI who underwent neurofeedback training. The patients’ ages ranged from 15 to 60 years. Eight patients were in the intervention group and participated in 20 neurofeedback sessions over four weeks. The control group, consisting of five patients, participated in the same sessions from the fifth to the eighth week of the project. The protocols incorporated beta and alpha coherence methods, with participants keeping their eyes open during each 50-minute session. Electrodes were placed on the FP1-T3 and Cz-Oz regions. However, the Wechsler Memory Scale (WMS-IV) and Continuous Attention Test (DAUF test) did not show a statistically significant improvement in short-term memory, long-term attention, and concentration performance [18]. 

In 2021, Arroyo-Ferrer [19] and colleagues compared neurofeedback with traditional cognitive rehabilitation methods. A 20-year-old patient with a brain injury and three healthy controls received NFB training based on theta band inhibition. All participants underwent eight 45-minute NFB training sessions over two weeks. Visual feedback was provided, and three different visual scenarios were used. The project lasted six weeks. The patient had a two-week rest period and participated in conventional rehabilitation during the final two weeks. The Brief Test of Attention (BTA) task measured attention and improved after neurofeedback and the traditional method. Short-term memory appeared to improve after conventional rehabilitation, measured by the Rey Osterrieth Complex Figure (ROCF) and the TAVEC-Verbal Learning Test Espana Complutense. In contrast, delayed memory showed improvement after NFB training [19]. 

Hyperbaric oxygen therapy for TBI 

Hyperbaric oxygen therapy (HBOT) is suggested as a potential treatment for traumatic brain injuries. This therapy, which combines pressure and an elevated oxygen concentration, leads to more oxygen dissolved in the blood, increasing blood oxygen levels and potentially enhancing cognitive abilities and the body's innate healing processes, thus reducing symptoms. This could provide a healing advantage for brain injuries. The dissolved oxygen can reach further into the injured brain tissue than typically possible, aiding healing. The outcome of this therapy can be an enhancement in cognitive abilities and a reduction in symptoms [20]. HBOT has been explored as a therapy for TBI and Post Traumatic Stress Disorder (PTSD), administered anywhere from 3 to 71 months post-injury for mild TBI and within 24 hours for moderate to severe TBI. Among people who experience mild TBI, HBOT has shown potential benefits [21]. In a Cochrane Review, the authors found seven eligible studies involving 571 people. The combined results suggest that HBOT reduces the risk of death and improves the level of coma; however, there is no evidence that these survivors have an improved outcome in terms of quality of life. It is possible, therefore, that the overall effect of hyperbaric oxygen is to make it more likely that people will survive with severe disability after such injuries. The authors conclude that the routine use of HBOT in brain-injured patients cannot be justified by the findings of this review [22]. 

A systematic review and dosage analysis were conducted on the efficacy of HBOT in Mild Traumatic Brain Injury Persistent Post-Concussion Syndrome (PPCS). The study found that patients treated with 40 HBOTs at 1.5 atmospheres absolute (ATA) showed statistically significant symptomatic and cognitive improvements [23]. A study published in PLOS suggested that repetitive HBOT improves outcomes in traumatic brain injury, indicating that protective long-term HBOT effects following brain injury are mediated by a pronounced remyelination in the ipsilateral injured cortex as substantiated by the associated recovery of sensorimotor function [24]. 

Retrospective analysis of patients suffering from chronic neuro-cognitive impairment from TBI treated with HBOT. The HBOT protocol included 60 daily HBOT sessions, five days per week. All patients had pre and post-HBOT objective computerized cognitive tests (NeuroTrax) and brain perfusion MRI. Ten post-TBI patients were treated with HBOT with a mean of 10.3±3.2 years after their injury. After HBOT, whole-brain perfusion analysis showed significantly increased cerebral blood flow and volume. Clinically, HBOT significantly improved the global cognitive scores (p = 0.007). The most prominent improvements were in information processing speed, visual-spatial processing, and motor skills indices. HBOT may induce cerebral angiogenesis, which improves perfusion to the chronically damaged brain tissue even months to years after the injury [25]. 

Fifteen patients afflicted with PPCS were treated with 60 daily HBOT sessions. Imaging evaluation was performed using Dynamic Susceptibility Contrast-Enhanced (DSC) and Diffusion Tensor Imaging (DTI) MR sequences. A cognitive evaluation was performed by an objective computerized battery (NeuroTrax). HBOT was initiated 6 months to 27 years (10.3 ± 3.2 years) from injury. After HBOT, DTI analysis showed significantly increased fractional anisotropy values and decreased mean diffusivity in white and gray matter structures. In addition, the cerebral blood flow and volume increased significantly. Clinically, HBOT significantly improved memory, executive functions, information processing speed, and global cognitive scores [25]. 

The trial population included 56 moderate TBI patients 1-5 years after injury with prolonged post-concussion syndrome (PCS). The HBOT effect was evaluated employing a prospective, randomized, crossover-controlled trial: the patients were randomly assigned to treated or crossover group and were evaluated at baseline and following 40 HBOT sessions; patients in the crossover group were evaluated three times: at baseline, following a 2-month control period of no treatment, and following subsequent 2-months of 40 HBOT sessions. The HBOT protocol included 40 treatment sessions (5 days/week), 60 minutes each, with 100% oxygen at 1.5 ATA. ‘‘Mindstreams’’ was used for cognitive evaluations, quality of life (QOL) was evaluated by the EQ-5D, and changes in brain activity were assessed by SPECT imaging. Significant improvements were demonstrated in cognitive function and QOL in both groups following HBOT, but no significant improvement was observed following the control period. SPECT imaging revealed elevated brain activity in good agreement with cognitive improvements. The researchers concluded that HBOT can induce neuroplasticity, leading to the repair of chronically impaired brain functions and improved quality of life in mild TBI patients with prolonged PCS at a late chronic stage [26]. 

A retrospective analysis was conducted of 154 patients suffering from chronic neurocognitive damage due to TBI who had undergone computerized cognitive evaluations pre-HBOT and post-HBOT treatment. The average age was 42.7±14.6 years, and 58.4% were men. All patients had documented TBI 0.3-33 years (mean 4.6±5.8, median 2.75 years) before HBOT. HBOT was associated with significant improvement in all cognitive domains, with a mean change in global cognitive scores of 4.6±8.5 (p<0.00001). The most prominent improvements were in-memory index and attention, with mean changes of 8.1±16.9 (p<0.00001) and 6.8±16.5 (p<0.0001), respectively. The most striking changes observed in brain single photon emission computed tomography images were in the anterior cingulate and the postcentral cortex, in the prefrontal areas, and in the temporal areas [27]. 

Combined neurofeedback and hyperbaric oxygen therapy treating TBI 

White et al. [4] reported that in 2014, a male, aged 26, suffered a severe TBI due to a car accident. The injury was a closed head wound on the left temporal region with a coup contrecoup impact on the frontal area. The patient underwent a craniotomy on the left side and remained in a coma for 26 days. Upon regaining consciousness, he was transferred to a treatment center specializing in brain injuries, where he received physical, speech, and occupational therapy. After eight months, he was discharged with significant issues related to speech, mobility, spasticity, cognition, and posttraumatic epilepsy. His parents then sought HBOT from a physician in Louisiana. After 165 HBOT sessions, the doctor recommended incorporating neurofeedback therapy. The patient began neurofeedback therapy in conjunction with HBOT in March 2019. This combined approach led to improvements in the plasticity and functionality of the injured areas, as well as correlated symptoms such as short-term memory, personality, language, and executive function. It also significantly reduced the frequency of seizures. Severe brain injuries often result in persistent deficits, with limited prospects for substantial recovery, underscoring the need for more research into long-term, effective neurological treatments. The findings suggest that a combination of HBOT and neurofeedback could be a promising treatment option for severe brain injuries and warrants further investigation [4]. White et al [4] appears to be the only study examining the treating of TBI with a combined neurofeedback and hyperbaric oxygen therapy. Our study aims to align with their efforts to fill this void. 

## Case presentation

On July 30, 2018, an accident occurred where a 33-year-old female runner was hit by a car moving at 40 mph. The impact was so severe that it resulted in her being thrown 30 feet away. She sustained serious injuries, including a TBI, and when found, she was unconscious. Emergency Medical Services (EMS) were called, and she was rushed to the emergency room. The patient had diagnosis S06.2X9D, diffuse TBI with loss of consciousness of unspecified duration, subsequent encounter. Substantial severe TBI trauma was on the left side of the head. The patient was in a coma for seven weeks, with three weeks in the Intensive Care Unit (ICU). She spent another seven months in intensive rehabilitation, and during that time, her caregivers asked about HBOT. Neurosurgeons, neurologists, and physical medicine physicians remarked to the patient's caregivers that HBOT was good for wounds, but the studies do not show that it is helpful. She regressed for approximately three weeks, swapped homes to be in a barrier-free home, and was there until the end of 2019. In October of 2021, her caregivers switched physicians and obtained a neuropsychiatrist who strongly recommended HBOT. The neuropsychiatrist commented that she had read the studies and noted that a thorough investigation of the research reveals that it does help many people. Her caregivers remarked that pure oxygen takes care of inflammation based on research and testimony, with little or no risk and tremendous upside potential. The patient's caregivers noted that HBOT helped her clear her thought process. The patient also continues in physical therapy six days a week. 

The patient had her first HBOT, q-EEG, and neurofeedback treatment on November 10, 2021.

On January 3, 2022, the patient reported sleeping well and having more extended, detailed conversations. Anger is diffusing, and there is increased meaning and understanding of everything. Things are better with her husband. The patient has goals of more conversation, accepting help, moving forward, and improving balance. HBOT maintenance was recommended two times per week and then re-reassessed. Neurofeedback treatments were recommended to continue for six to eight more sessions, two times per week. The patient will continue HBOT maintenance and neurofeedback maintenance. 

As of December 12, 2023, the patient has received 195 neurofeedback treatments and has self-reported improvements with impulsiveness, aggressiveness, agitation, difficulty falling asleep, negative thoughts, pain awareness, happiness, being organized, clear thinking, reaction time, attention and concentration, having her act together, reading, motivation, ability in tasks requiring steps, pain threshold, memory, body awareness, energy, and talkativeness.

Method of intervention: Neurofeedback 

Instruments 

Discovery 24-channel EEG amplifier (BrainMaster Technologies, Bedford, USA): The Discovery 24-channel EEG amplifier [28], a physiological monitoring and feedback system, was used. It offers monitoring and feedback of brain signals that include the measurement of EEG, direct current, and slow cortical potentials (DC/SCP). The system features 24 channels of EEG Biofeedback recording, including 22 channels connected to a standard electrode cap, plus two channels of differential inputs with separate references, useful for monitoring any of a wide range of EEG or related potentials. It provides 1024 samples per second on all channels, with 24-bit resolution and an amplifier bandwidth from DC (0.000 Hz) to (80 Hz). 

A lightweight, portable device, it is ideal for laptop computers for remote or home training and clinical or laboratory use.  The unit is entirely powered by the USB interface, eliminating the need for batteries while maintaining client safety [28]. 

WaveGuard Connect-19 Channel EEG Cap. (Bio-Medical Instruments, Clinton Township, USA): WaveGuard Connect-19 Channel EEG Cap. [29] uses soft silicone electrode cups and features hidden wiring and high-density connectors, making the use and maintenance of the caps quick, safe, and accessible. The cap is perfect for routine diagnostics where good signal quality is crucial for valid assessment. Tin has been widely used in clinical and research areas and has good longevity and signal quality. It is the most common electrode sensor material in clinical applications as it has proved very reliable in AC-coupled clinical recordings with a frequency range higher than 0.5 Hz. The electrode sensors are made of pure, solid tin, which provides the user with optimal signal quality. All cap electrodes are pre-positioned according to the international 10/20 system, and applying the caps is easy, with the preparation time for each patient significantly reduced compared to placing each electrode individually. The entire procedure, from preparation and gelling to recording, is achieved in less than 10 minutes [29]. 

WaveGuard Connect caps are compatible with all significant EEG amplifiers used in clinical environments, such as NicoletOne and Mitsar. The EEG caps default with a D-SUB 25 connector for a quick and straightforward connection. A D-SUB to single-point touch-proof adapter can be additionally purchased for interaction with other third-party amplifier headboxes [29]. 

WinEEG (Mitsar Brain Diagnostic Solutions, St. Petersburg, Russia): *WinEEG *is an advanced q-EEG research-grade software for EEG and Event-Related Potentials (ERP) data post hoc processing and analysis. Native Mitsar-EEG format files and international European Data Format (EDF) and EDF+ files could be imported for processing. WinEEG supports up to 256 recording channels, including event markers and triggers for ERP studies. A flexible EEG review allows fast display of the EEG on the computer monitor and immediate access to any part of the recording. User-definable labels may be used to mark EEG recordings for quick access to different parts of recordings. Artifacts correction procedures are based on Infomax ICA decomposition of raw EEG and special filtering, helping to increase the quality of EEG records. Eye movements are suppressed by ICA Artifacts correction by ICA templates Artifacts detection by threshold [30]. 

WinEEG software conducts “Event-Related Potentials Wavelet Analysis.” WinEEG software includes multichannel spectral analysis, brain mapping, and coherence. Power spectra and coherence can be computed for any selected part of the recorded EEG. Different spectra parameters computed for predefined frequency band ranges can be displayed as histograms, maps, and tables. The band range ratios like Theta/Beta and asymmetry maps are available with spectra computation. The spectra data can be exported to other applications (standard statistical packages such as STATISTICA (TIBCO Software, Palo Alto, USA) or SPSS (IBM Corp., Armonk, USA)) using the American Standard Code for Information Interchange (ASCII) format for future statistical analysis [30]. 

WinEEG is integrated with Low Resolution Electromagnetic Tomography (LORETA) software. LORETA software can perform sources and Spectra Power distribution with 3D mapping of data to which mapped data is transferred automatically. Also, you can perform LORETA for Independent Component Analysis (ICA) components and averaged Spikes. The spectra, coherence, Event-Related Potentials (ERP), Event-Related Desynchronization (ERD), etc., can be processed automatically for a batch of recordings the user selects. The results of processing will be automatically stored in a built-in database. Grand average files of Spectra and ERP data could be created and stored in the database. Comparison of subject Fast Fourier Transform (FFT) Power Spectra and ERP data to grand average files and pre- and post-recording comparison if available [30]. 

Built-in equivalent dipole localization algorithm helps to identify a brain position of the source of paroxysmal activity. All patient information, including EEG waveform and video, can be saved to a built-in database or written on CD. A built-in database helps to automatically search, record, and provide automatic data processing, data averaging, and exporting. In addition, all data can be stored and reviewed on any Windows-based PC. Raw or proceed EEG and ERP data can be exported in different formats such as ASCII, EDF and EDF+, and others. EEG spectra, coherence, ERP, ERD, and parameters of task performance can be exported to an ASCII file automatically for the collection of recordings selected by the user [30]. 

Participant and procedure 

The patient agreed to participate in this study, and her caregivers signed an informed consent for administering the neurofeedback and HBOT and for the research use of the qEEG data reports generated. The patient was seen and given the neurofeedback treatments at The Oxford Centers (ORC; Brighton and Troy, Michigan, U.S.A). The Oxford Centers are outpatient facilities that provide various services for several biopsychosocial conditions. 

A baseline (Pretest) qEEG was acquired from the patient on November 10, 2021, before the beginning of the neurofeedback treatments. For the gathering of the qEEG data, the patient was fitted with the Channel EEG Cap [31] for scalp electrodes with 19 channels placed relative to the International 10/20 System (Fp1, Fp2, F7, F3, Fz, F4, F8, T7, C3, Cz, C4, T8, P7, P3, Pz, P4, P8, O1, and O2.), including ear connectors. For 20-30 minutes, EEG action potentials emitted from the 19 channels were accumulated and gathered using the Discovery 24-channel EEG amplifier [30]. Impedances of < 10KOhms were maintained. EEG output was uploaded into WinEEG [32] for data analysis, with the artifacts identified and removed. EEG results within WinEEG indicated resulting power in microvolts squared (uV²) and hertz (Hz) readings. Delta, Theta, Alpha, Beta-1, Beta-2, and Gamma were reported. 

Neurofeedback protocol

The neurofeedback training protocol was primarily directed towards the left hemisphere of the brain, which was the site of the injury. However, the protocol also took into account the potential impact on interconnected regions of the brain due to the complex and integrated nature of brain networks. The neurofeedback approach was tailored based on the patient’s qEEG readings. The specific objective of the neurofeedback was to augment the power of alpha and beta brainwaves while concurrently diminishing the amplitude of theta or delta waves. This strategy was based on the premise that such modifications in brainwave activity could potentially facilitate cognitive recovery.

The patient participated in the neurofeedback training with a frequency of two to three sessions per week. Each session spanned a duration of 30-60 minutes. The schedule was flexible, accommodating intermittent breaks to account for holidays and personal commitments. Each individual session incorporated the use of computer games, animations, and sounds that were responsive to the patient’s brainwave activity. This interactive setup provided real-time feedback, guiding the patient to consciously alter their brainwave activity through visual or auditory stimuli. The training activities were designed to engage cognitive tasks that demanded attention, memory, and executive function. The purpose of these tasks was to reinforce desired brainwave patterns, thereby promoting the reestablishment of healthy cognitive processes. 

Posttest qEEG

On June 6, 2024, a posttest qEEG was acquired from the patient to ensure consistency and comparison with previous measurements. The data collection process involved fitting the patient with the Channel EEG Cap, which features 19 channels positioned according to the International 10/20 System. These positions included Fp1, Fp2, F7, F3, Fz, F4, F8, T7, C3, Cz, C4, T8, P7, P3, Pz, P4, P8, O1, and O2, along with ear connectors to enhance signal fidelity.

EEG action potentials from the 19 channels were continuously recorded using the Discovery 24-channel EEG amplifier. Throughout the recording session, electrode impedances were carefully maintained below 10 KOhms to ensure high-quality data acquisition.

The collected EEG data were then uploaded to WinEEG software for detailed analysis. This process included the identification and removal of artifacts to ensure the accuracy of the resulting data. 

The analysis provided detailed information on various brainwave activities, including Delta, Theta, Alpha, Beta-1, Beta-2, and Gamma frequencies. These metrics offered valuable insights into the patient's neural function and allowed for a comprehensive assessment of changes following the neurofeedback and HBOT treatments initiated in 2021.

qEEG Codes for Power (uV2) and Frequency (Hz) 

In a qEEG, these abbreviations describe various parameters and measurements related to brainwave activity. The meaning of each abbreviation is as follows.

DuV² (Delta microvolts squared): This represents the power of the delta waves (0.5-4 Hz frequency range) measured in microvolts squared. Delta waves are associated with deep sleep and restorative processes. The patient achieved statistically significant improvement in this area.

Dhz (Delta Hertz): This represents the frequency of delta waves measured in Hertz. Delta waves are associated with deep sleep and restorative processes.

TuV² (Theta microvolts squared): This represents the power of the theta waves (4-8 Hz frequency range) measured in microvolts squared. Theta waves are linked to meditation, early stages of sleep, learning, and memory processes.

Thz (Theta Hertz): This represents the frequency of theta waves measured in Hertz. Theta waves are linked to meditation, early stages of sleep, learning, and memory processes. The patient achieved statistically significant improvement in this area. 

AuV² (Alpha microvolts squared): This represents the power of the alpha waves (8-13 Hz frequency range) measured in microvolts squared. Alpha waves are associated with relaxation, calm, wakefulness, and attention. The patient achieved statistically significant improvement in this area. 

Ahz (Alpha Hertz): This represents the frequency of alpha waves measured in Hertz. Alpha waves are associated with relaxation, calm, wakefulness, and attention. The patient achieved statistically significant improvement in this area. 

BuV² (Beta microvolts squared): This represents the power of the beta waves (13-30 Hz frequency range) measured in microvolts squared. Beta waves are linked to active thinking, focus, and anxiety. 

Bhz (Beta Hertz): This represents the frequency of beta waves measured in Hertz. Beta waves are linked to active thinking and focus. 

B2uV² (Beta2 microvolts squared): This represents the power of the higher frequency range of beta waves (20-30 Hz) measured in microvolts squared. Beta2 waves are often associated with heightened alertness and intense focus. The patient achieved statistically significant improvement in this area. 

B2hz (Beta2 Hertz): This represents the frequency of the higher frequency range of beta waves measured in Hertz. Beta2 waves are often associated with heightened alertness and intense focus.

GuV² (Gamma microvolts squared): This represents the power of the gamma waves (30-100 Hz frequency range) measured in microvolts squared. Gamma waves are associated with higher cognitive functions like learning, memory, and information processing. The patient achieved statistically significant improvement in this area. 

Ghz (Gamma Hertz): This represents the frequency of gamma waves measured in Hz. Gamma waves are associated with higher cognitive functions like learning, memory, and information processing. 

Again, these parameters help analyze the brain's electrical activity, giving insights into various cognitive and neural functions and identifying abnormalities in brain activity. 

Method of intervention: Hyperbaric Oxygen Therapy (HBOT) 

From November 2021 to June 2024, a Class B monoplace hyperbaric chamber (Sechrist 3300H, Sechrist Industries, Inc., Anaheim, California, United States) was employed at The Oxford Center in Brighton, United States, to provide HBOT to the patient diagnosed with severe TBI. The chamber was filled with medical-grade oxygen pressurized between 1.5 and 2.0 ATA at a 1-2 psi/min speed, maintaining an average oxygen concentration of 100%. This treatment was administered up to five times a week. Trained hyperbaric technicians closely monitored the patient for any adverse reactions. After each session, the chamber was depressurized from 1 to 2 psi/min back to 1.0 ATA. 

Prior to administration, a Certified Hyperbaric Technician (CHT) conducted a pre-treatment screening, which included setting treatment goals, reviewing the patient's medical history, and discussing potential benefits and risks. The patient was also taught how to equalize her ear pressure, like flying on a commercial airplane, to avoid discomfort during the treatment. 

The patient was given hospital scrubs to wear during the session and was asked to remove any metal objects, such as jewelry, glasses, dentures, contact lenses, and other items that could be damaged by the high-pressure oxygen environment. The patient was then placed in the chamber, which was filled with pure medical-grade oxygen, and the chamber was sealed. The CHT gradually increased the pressure in the chamber while maintaining communication with the patient via an intercom system. Treatments lasted between 30 minutes and two hours. Following the treatment, the chamber was slowly depressurized, and the patient could leave. The patient and caregiver were then advised to hydrate the patient and rest before resuming her regular activities. 

Disability measures 

Three clinicians and one caregiver completed the Disability Rating Scale (DRS), assessing retrospectively from 2021 for the patient’s status in 2024, and The Glasgow Outcome Scale (GOSE) for status in 2024. 

*The Disability Rating Scale (DRS)* [31,32], used to assess individuals with moderate and severe TBI, was developed to functionally evaluate a TBI patient from coma to community reintegration. The scale rates the effects of injury and estimates recovery duration [30,31]. The rating provides insight into cognitive impairment and measures eight areas of functioning in four categories: (1) Consciousness: eye-opening, verbal response, motor response; (2) Cognitive ability: feeding, toileting, grooming; (3) Dependence on others, and (4) Employability. Each area of functioning is rated on a scale of 0 to either 3 or 5, with the highest scores representing a higher level of disability. The maximum cumulative score is 29 (representing an extreme vegetative state), and the minimum cumulative score is 0 (representing a person without a disability) [31,32]. 

*The Glasgow Outcome Scale Extended (GOSE) *[33,34] measures global disability and recovery with an eight-level indicator of overall functional outcomes: (1) Dead, (2) Vegetative State: the patient exhibits reflex responses and periods of spontaneous eye opening but remains unaware, (3) Lower Severe Disability: characterized by the patient’s dependence on others for daily support due to a combination of mental and physical disabilities. If the patient cannot be left alone at home for over 8 hours, they are considered at the lower level of Severe Disability. (4) Upper Severe Disability: if the patient can be left alone at home for over 8 hours, they are considered at the upper level of Severe Disability. (5) Lower Moderate Disability: patients have some disability, such as aphasia, hemiparesis, epilepsy, memory deficits, or personality changes, but can care for themselves. They are independent at home but require assistance outside. If they cannot return to work, they are considered at the lower level of Moderate Disability. (6) Upper Moderate Disability: if patients can return to work, even with special arrangements, they are considered at the upper level of Moderate Disability. (7) Lower Good Recovery: patients have resumed everyday life and can work, even if they have not achieved their pre-injury status. Some patients may have minor neurological or psychological deficits. If these deficits are disabling, they are considered at the lower level of Good Recovery. (8) Upper Good Recovery: if these deficits are not disabling, patients are considered at the upper level of Good Recovery [33,34].

Statistical analysis and disability assessment raters

Evaluation through the DRS and the GOSE provides insights into the complex trajectory of recovery and long-term functional outcomes following TBI. Analyzing the patient's progression from 2021 (Pretest) to 2024 (Posttest) offers a comprehensive understanding of the evolving cognitive, physical, and psychosocial dimensions post-injury. 

Quantitative data analysis was carried out utilizing the Wilcoxen Rank Sum Test. This analysis used the Statistical Package for the Social Sciences (SPSS) software, version 29.0 (IBM Corp., Armonk, USA). The evaluation process involved four distinct raters who contributed their assessments. 

These raters included the patient’s neuropsychiatrist, a specialist in neurology and psychiatry who diagnoses and treats neurological conditions with psychiatric symptoms. The second rater was the patient’s husband, who also served as the caregiver and provided necessary care and support to the patient. 

The third rater was a neurofeedback technician. Neurofeedback technicians are trained professionals who use real-time displays of brain activity, commonly EEG to teach self-regulation of brain function. They play a crucial role in administering neurofeedback therapy, which can help improve the patient’s mental state. 

The fourth rater was an HBOT technician. HBOT technicians operate the HBOT chambers. Their assessment would be particularly valuable in understanding the patient’s response to HBOT treatment. 

Each of these raters provided their unique perspectives and expertise, contributing to a more holistic and comprehensive data gathering. Their combined assessments would provide valuable insights into the patient’s condition and response to treatment. 

qEEG and measurement outcomes Pre (2021) and Post (2024)

**Figure 1:**
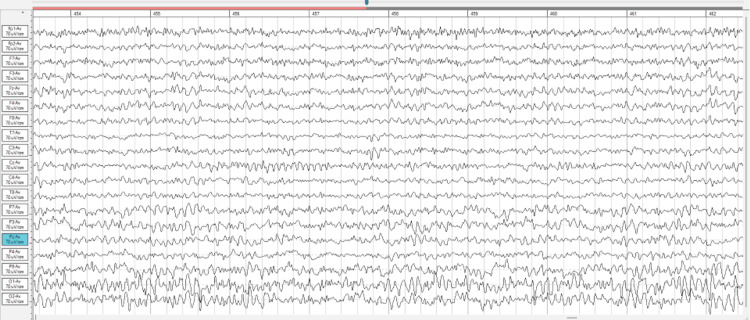
Raw qEEG for Pretest (2021) qEEG=Quantitative electroencephalogram Eyes open Pre EEG 454-461 epoc 70uV Gain 30mm/s Speed 0.1 (1.6Hz) Low Cut 50 High Cut 45-55 Notch Hz

Figure 1 below indicates significant slowing within the posterior region. High amplitude alpha is seen along with cross-frequency coupling of delta activity. Beta spindling is seen within the CZ region. This pattern is often associated with 'cortical irritability,' viral or toxic encephalopathies, and epilepsy. This abnormal beta manifests as waxing and waning spindles over the impacted cortex and can be correlated with dysregulated vagal tone. The Cz region beta spindling is activating at 15 Hz in one epoch, equivalent to approximately 60 microvolts. It typically presents a high-voltage beta activity that can occasionally surpass 20 microvolts.

**Figure 2:**
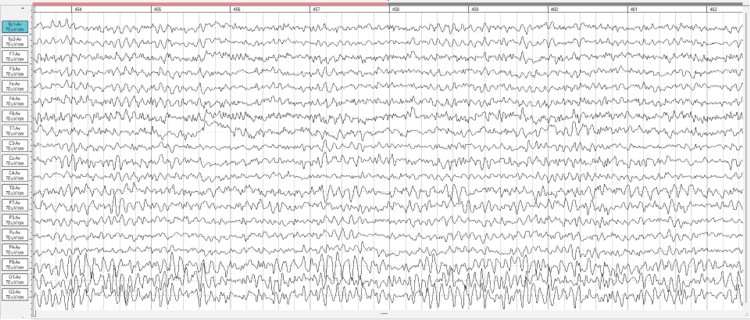
Raw qEEG for Posttest (2024) qEEG=Quantitative Electroencephalogram Post Neurofeedback and HBOT qEEG activity showing significantly less Delta activity in the posterior region.

The posttest scan in Figure 2 below indicates increased activation of the "default mode network". This network is said to be correlated with inward thought processing and consolidation of experiences, to make sense of personal experiences and memories [35]. The uV² shows high power of spectral density within the P8 region which is said to be a part of the default mode network, but also known to be correlated with a trauma history. This could lead to a hypothesis that as the patient's awareness increased, her memory of the event and current abilities came to her conscious, and thus is working to consolidate these experiences. As the patient's brain was given the chance to reorganize and stabilize, there was an increase in awareness of her current situation and a strong motivation to further her progress.

**Figure 3:**
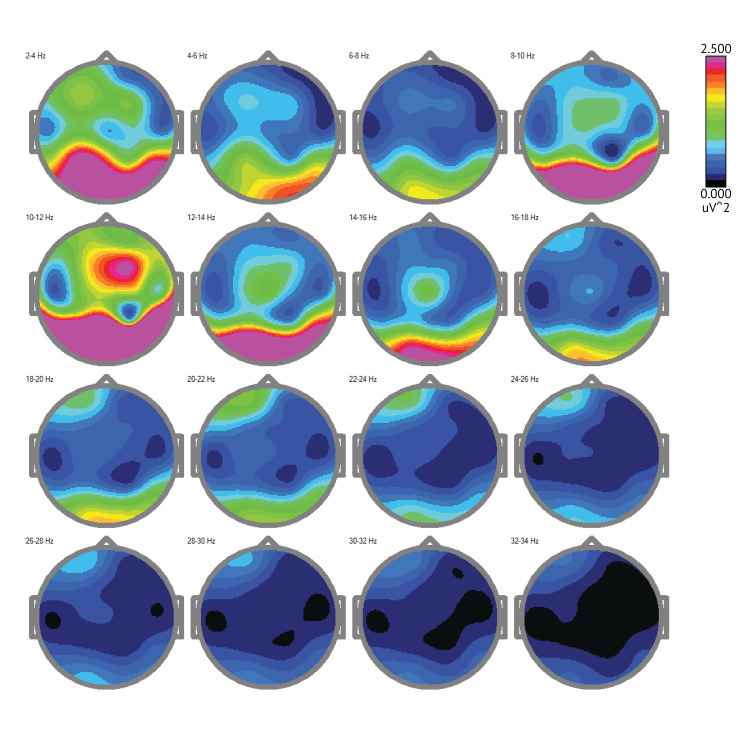
Pretest Brain Map (2021): Initial Assessment of Neural Structures and Functions

The image labeled as Figure 3 below demonstrates this patient's severe TBI which results in increased slow wave (delta and theta) activity and decreased fast wave (alpha, beta, and gamma) activity. This pattern reflects disrupted brain function and impaired cognitive processing. 2-4 Hz (Delta waves): High amplitude delta waves indicate severe brain injury. These slow waves are often present during deep sleep but can dominate in brain injury, indicating disrupted consciousness. 4-6 Hz (Theta waves): Increased theta activity indicates brain dysfunction. It is normal during light sleep, but excessive theta waves when awake can suggest cognitive impairment. 6-8 Hz: Increased theta activity similar to the 4-6 Hz range. 8-10 Hz (Alpha waves): Reduced alpha activity, as observed with this patient, is common after TBI. Alpha waves are associated with a relaxed, awake state, and a decrease indicates impaired brain function. 10-12 Hz: Reduced alpha activity, similar to the 8-10 Hz range. 12-14 Hz (Low Beta waves): Beta waves are associated with active thinking and focus. Decreased activity in this range indicates impaired cognitive processing. 14-16 Hz: Similar to the 12-14 Hz range, low beta waves are reduced. 16-18 Hz: Decreased beta activity can be observed. 18-20 Hz (Mid Beta waves): Lower levels of beta activity, common in TBI, indicate difficulties with active thinking and focus. 20-22 Hz: Continued low beta activity. 22-24 Hz: Decreased beta wave activity persists. 24-26 Hz (High Beta waves): Reduced high beta activity indicates impaired brain function and cognitive difficulties. 26-28 Hz: Low high beta activity similar to the 24-26 Hz range. 28-30 Hz (Gamma waves): Gamma waves are associated with higher cognitive functioning. Reduced gamma activity can indicate severe impairment in cognitive processing. 30-32 Hz: Continued low gamma activity. 32-34 Hz: Almost no detectable activity, indicating severe brain dysfunction.

After 31 months of treatment, on June 5, 2024, at posttest, 19 scalp electrode placement sites outcomes were reported for Microvolts squared (uV²) outcomes. Post-treatment qEEG results showed small to moderate average effects on 19 scalp electrode placement site outcomes (uV2 g=.339). Statistically significant p-values (p<.05) were reported at T7, Cz, and T8. This indicates significant progress in the patient's recovery journey. For a detailed report of the 19 scalp electrode placement sites outcomes in Microvolts squared (uV²) outcomes, please see Table 1 below.

**Table 1:** 19 Channel Scalp Electrode Placement Sites-Microvolts Squared (uV²)-Pre (2021)-Post (2024). *Wilcoxen Rank Sum Test; ** ES = Effect Size-Hedges' g; Hedges g Criteria; ±0.20=Low Effect; ±0.50=Moderate Effect; ±0.80=Large Effect; SD=Standard Deviation

Channel	Mean	n	SD	Median	p-value*	ES**	ES (95%CI)
Fp1AvPre	2.402	6	1.175	2.150	0.116	0.611	-0.178,1.356
Fp1AvPost	1.128	6	1.586	0.575			
Fp2AvPre	1.015	6	0.610	0.835	0.345	-0.092	-0.763,0.587
Fp2AvPost	1.173	6	1.974	0.390			
F7AvPre	1.762	6	0.659	1.670	0.345	0.200	-0.493,0.874
F7AvPost	1.308	6	1.881	0.600			
F3AvPre	1.850	6	0.806	1.790	0.345	0.389	-0.340, 1.084
F3AvPost	1.443	6	1.545	0.915			
FzAvPre	1.682	6	1.267	1.375	0.344	0.241	-0.459,0.918
FzAvPost	1.495	6	1.839	0.875			
F4AvPre	1.725	6	1.386	1.250	0.345	0.282	-0.425,0.963
F4AvPost	1.510	6	1.777	0.920			
F8AvPre	1.068	6	0.894	0.835	0.463	-0.363	-1.054,0.360
F8AvPost	1.717	6	2.367	0.860			
T7AvPre	0.652	6	0.309	0.615	0.046	-0.541	-1.267,0.228
T7AvPost	1.512	6	1.609	1.035			
C3AvPre	1.49	6	0.750	1.490	0.249	0.444	-0.298,1.150
C3AvPost	1.108	6	1.350	0.655			
CzAvPre	1.563	6	1.187	1.175	0.046	-0.414	-1.114,0.321
CzAvPost	2.460	6	2.898	1.410			
C4AvPre	1.160	6	0.831	0.975	0.249	0.400	0.332,1.097
C4AvPost	0.950	6	1.175	0.565			
T8AvPre	0.907	6	0.693	0.745	0.028	-0.524	-1.246,0.240
T8AvPost	2.948	6	3.944	1.600			
P7AvPre	2.710	6	2.182	1.925	0.345	0.128	-0.555,0.799
P7AvPost	2.485	6	3.444	1.165			
P3AvPre	2.877	6	2.251	2.115	0.075	0.652	-0.150,1.408
P3AvPost	1.810	6	2.639	0.810			
PzAvPre	2.192	6	1.952	1.480	0.345	0.082	-0.597,0.753
PzAvPost	2.058	6	2.933	0.990			
P4AvPre	1.375	6	0.847	1.105	0.674	-0.226	-0.902,0.471
P4AvPost	1.775	6	2.159	1.045			
P8AvPre	4.188	6	3.883	2.985	0.753	-0.337	-1.025,0.381
P8AvPost	8.512	6	14.567	2.440			
O1AvPre	6.950	6	8.082	4.310	0.345	-0.200	-0.875,0.492
O1AvPost	8.625	6	15.017	2.405			
O2AvPre	6.128	6	7.103	3.875	0.917	-0.317	-1.002,0.396
O2AvPost	10.657	6	19.002	2.720			

After 31 months of treatment, on June 5, 2024, at the posttest, qEEG results showed small to moderate average effects on 19 scalp electrode placement site outcomes (Hz g=.333). This indicates noteworthy progress in her recovery journey. Please refer to Table 2 below for more detailed results. Please see the Appendices for the qEEG 19 channel scalp electrode placement sites dataset.

**Table 2:** 19 Channel Scalp Electrode Placement Sites-Hertz (Hz)-Pre (2021)-Post (2024) *Wilcoxen Rank Sum Test; ** ES = Effect Size-Hedges' g Hedges g Criteria: ±0.20=Low Effect; ±0.50=Moderate Effect; ±0.80=Large Effect SD=Standard Deviation

Hertz (Hz)	Mean	n	SD	Median	p-value*	ES**	ES (95%CI)
Fp1AvPre	14.730	6	11.281	15.260	0.588	0.211	-0.483,0.886
Fp1AvPost	13.957	6	10.280	12.210			
Fp2AvPre	13.508	6	10.757	12.330	0.461	-0.338	-1.026,0.380
Fp2AvPost	14.078	6	10.367	12.210			
F7AvPre	14.323	6	10.960	15.260	0.752	0.067	-0.610,0.738
F7AvPost	14.080	6	9.954	12.210			
F3AvPre	13.468	6	10.952	11.965	0.461	0.267	-0.432,0.953
F3AvPost	13.267	6	10.450	12.210			
FzAvPre	13.428	6	10.608	12.330	0.465	0.354	-0.368,1.044
FzAvPost	12.467	6	11.094	9.570			
F4AvPre	13.427	6	10.692	12.330	0.997	-0.258	-0.936,0.445
F4AvPost	13.875	6	10.121	12.210			
F8AvPre	13.428	6	10.741	12.330	0.465	-0.329	-1.016,0.387
F8AvPost	13.997	6	10.166	12.210			
T7AvPre	13.387	6	10.774	12.085	0.273	-0.380	-1.074,0.347
T7AvPost	13.9967	6	9.941	12.085			
C3AvPre	13.3067	6	10.711	11.965	0.465	-0.428	-1.131,0.310
C3AvPost	14.2817	6	10.747	11.600			
CzAvPre	13.3467	6	10.451	12.330	0.705	-0.225	-0.901,0.472
CzAvPost	13.7133	6	10.295	11.600			
C4AvPre	13.2267	6	10.492	12.210	0.144	-0.539	-1.265,0.229
C4AvPost	13.63	6	10.846	12.085			
T8AvPre	13.227	6	10.492	12.210	0.357	-0.370	-1.063,0.342
T8AvPost	13.917	6	10.544	11.600			
P7AvPre	13.267	6	10.523	12.210	0.357	-0.387	-1.083,0.342
P7AvPost	13.957	6	10.430	11.720			
P3AvPre	13.267	6	10.480	12.330	0.285	-0.367	-1.060,0.357
P3AvPost	13.875	6	10.121	12.210			
PzAvPre	13.267	6	10.480	12.330	0.104	-0.484	-1.198,0.269
PzAvPost	14.034	6	10.210	12.330			
P4AvPre	13.347	6	10.633	12.330	0.465	-0.331	-1.018,0.386
P4AvPost	13.917	6	10.0578	12.210			
P8AvPre	13.145	6	10.51982	11.965	0.593	-0.302	-0.986,0.408
P8AvPost	13.672	6	10.214	11.600			
O1AvPre	13.348	6	10.725	12.210	0.655	-0.313	-0.997,0.400
O1AvPost	13.875	6	10.121	12.210			
O2AvPre	13.307	6	10.782	11.965	0.257	-0.369	-1.062,0.355
O2AvPost	13.915	6	10.298	12.085			

**Figure 4:**
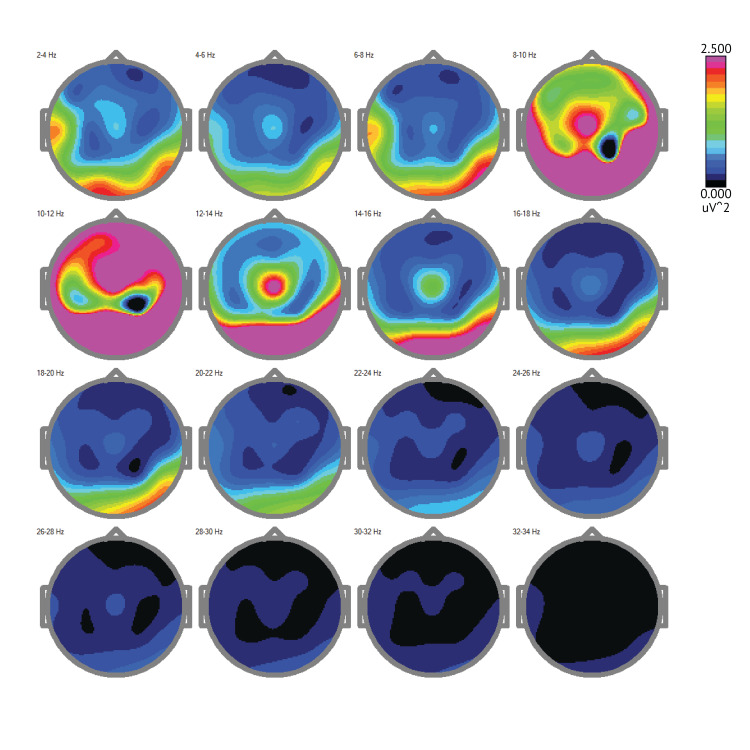
Posttest Brain Maps (2024): Insights into Neural Activity and Connectivity

As indicated in Figure 4 below, overall, the patient's posttest brain map shows increased alpha, beta, and gamma wave activity with reduced delta and theta waves, indicating significant recovery and improved brain function after 31 months of therapy. 2-4 Hz (Delta waves): Some reduction in high amplitude delta waves, indicating partial improvement in brain activity but still some regions with slow wave activity. 4-6 Hz (Theta waves): Decreased theta activity, showing improved cognitive function and lessened brain dysfunction. 6-8 Hz: Continued reduction in theta activity, indicating ongoing recovery. 8-10 Hz (Alpha waves): Increased alpha activity, suggesting a more relaxed and awake state, reflecting better cognitive function. 10-12 Hz: Enhanced alpha waves, further indicating improved brain function. 12-14 Hz (Low Beta waves): Increased low beta activity, reflecting better focus and cognitive processing. 14-16 Hz: Continued improvement in low beta waves. 16-18 Hz: Further increase in beta activity, showing improved mental activity. 18-20 Hz (Mid Beta waves): Enhanced mid-beta waves, indicating improved cognitive function. 20-22 Hz: Continued increase in mid-beta activity. 22-24 Hz: Further enhancement in beta wave activity. 24-26 Hz (High Beta waves): Increased high beta activity, indicating better cognitive processing and brain function. 26-28 Hz: Continued improvement in high beta waves. 28-30 Hz (Gamma waves): Increased gamma activity, indicating improved higher cognitive functions. 30-32 Hz: Continued enhancement in gamma waves.32-34 Hz: Significantly improved gamma activity, reflecting higher cognitive recovery.

Please see Table 3 below for qEEG Frequency Band Parameters Outcomes - Pre (2021) - Post (2024). Please see the Appendices for the qEEG frequency band parameters outcomes - pre (2021) - post (2024) dataset.

**Table 3:** qEEG Frequency Band Parameters Outcomes – Pre (2021) - Post (2024) *Wilcoxen Rank Sum Test; ** ES = Effect Size-Hedges' g Hedges’ g Criteria: ±0.20 = Low Effect; ±0.50 = Moderate Effect; ±0.80 = High Effect uV²=Microvolts Squared; Hz=Hertz; SD=Standard Deviation DuV²=Delta Microvolts Squared, DHz=Delta Hertz, TuV²=Theta Microvolts Squared, THz= Theta Hertz, AuV²=Alpha Microvolts Squared, AHz=Alpha Hertz, BuV²=Beta Microvolts Squared, BHz=Beta Hertz, B2uV²=Beta 2 Microvolts Squared, B2Hz=Beta 2 Hertz, GuV²=Gamma Microvolts Squared, GHz=Gamma Hertz.

Frequency Band	Mean	n	SD	Median	p-value*	ES**	ES (95%CI)
PreDuV²	2.187	19	1.353	1.93	0.001	0.928	0.394,1.445
PostDuV²	1.112	19	0.657	0.94			
PreDHz	1.460	19	0.000	1.46	0.157	-0.32	-0.758,0.127
PostDHz	1.538	19	0.233	1.46			
PreTuV²	1.272	19	0.780	1.02	0.095	0.353	-0.097,0.794
PostTuV²	1.068	19	0.665	0.83			
PreTHz	3.960	19	0.128	3.91	<0.001	-2.046	-3.288,-1.507
PostTHz	6.832	19	1.159	7.32			
PreAuV²	5.878	19	6.126	3.25	<0.001	-0.673	-1.148,-0.182
PostAuV²	11.809	19	13.798	6.12			
PreAHz	10.471	19	0.313	10.50	0.004	0.751	0.248,1.293
PostAHz	10.152	19	0.517	10.50			
PreBuV²	1.898	19	1.335	1.50	0.235	0.134	-0.300,0.565
PostBuV²	1.787	19	1.709	1.06			
PreBHz	14.575	19	1.841	13.92	0.141	0.333	-0.155,0.773
PostBHz	13.933	19	0.055	13.92			
PreB2uV²	1.825	19	1.0776	1.45	0.001	0.649	0.163,1.121
PostB2uV²	1.155	19	0.694	0.94			
PreB2Hz	20.112	19	0.574	19.78	0.291	-0.22	-0.653,0.220
PostB2Hz	20.382	19	0.881	20.26			
PreGuV²	0.737	19	0.516	0.52	<0.001	0.74	0.239,1.225
PostGuV²	0.334	19	0.154	0.30			
PreGHz	30.185	19	0.373	30.27	0.304	-0.251	-0.686,0.190
PostGHz	30.337	19	0.334	30.27			

## Discussion

Neurofeedback outcomes pretest (2021) and posttest (2024) qEEG 

Our findings revealed large, moderate, and small effect sizes (Tables 1-3) in several key qEEG 10/20 electrode regions, including FP1, C3, C4, Pz, T7, and T8, indicating notable improvements in brain function, cognition, and communication. These positive changes can be attributed to enhanced neuroplasticity and cerebral blood flow facilitated by neurofeedback and HBOT.

Improvements in Specific EEG Electrode Regions

Fp1 (Frontal Pole): The FP1 electrode in the frontal pole region is associated with executive functions, attention, and emotional regulation. Significant improvements in this area suggest enhanced cognitive control, better decision-making abilities, and improved emotional stability. Neurofeedback likely regulated neural oscillations, promoting more efficient communication between frontal regions and other brain parts.

C3 and C4 (Central Regions): Electrodes C3 and C4 are situated over the sensorimotor cortex and are crucial for motor control and sensory processing. The observed enhancements in these regions imply better motor function and coordination and improved sensory integration. This is particularly relevant for a TBI patient, as these improvements can lead to better physical rehabilitation outcomes and a higher quality of life. HBOT may have played a role in reducing inflammation and promoting neuronal repair in these areas, further supporting motor recovery.

Pz (Parietal Region): The Pz electrode is positioned over the parietal cortex, integrating sensory information and spatial awareness. Positive changes in this region indicate enhanced sensory processing and spatial orientation, leading to better daily functioning and independence. Improved EEG communication in Pz suggests that neurofeedback facilitated the synchronization of parietal networks, contributing to more efficient processing of sensory inputs.

Additionally, the Pz region is known for the posterior dominant rhythm (PDR), which is typically observed as alpha wave activity (8-12 Hz) when the brain is at rest but awake, with eyes closed,

increased cognitive abilities, such as better memory, attention, and overall cognitive processing. This rhythmic activity is crucial for cognitive functions, reflecting a well-organized and efficient neural network. Therefore, improvements in the Pz region and the associated PDR could signify a restoration of normal brain rhythms and enhanced cognitive capabilities.

T7 and T8 (Temporal Regions): Electrodes T7 and T8 are located over the temporal lobes, critical for memory, language, and auditory processing. Significant improvements in these regions suggest better memory retention, language comprehension, and aural discrimination. The combination of neurofeedback and HBOT likely promoted neurogenesis and synaptic plasticity in the temporal lobes, leading to these cognitive enhancements.

Theta shifts: microglial activation and cognition

Neurons not connected to a network fire at 3-6hz. This range of frequencies is correlated with microglial activation. According to Shao et al., microglia are primarily immune cells in the central nervous system (CNS) and play a crucial role in the brain’s response to injury and disease [36,37]. Following a TBI, microglia become activated, changing their morphology and function. This activation can contribute to both neuroprotective and neurotoxic outcomes. Activated microglia can release pro-inflammatory cytokines and reactive oxygen species, contributing to chronic inflammation and secondary injury. When looking at the pre and post scans, the average hz range of the Theta band was 3.96hz [36,37]. This data correlates to overactivation of the microglia cells. On a functional level, this indicates that all her theta band waves were activating apart from a network, which is an indicator of white matter damage. When neurons fire outside of these networks, it can lead to a wide range of neurological, cognitive, and behavioral issues. The patient’s post-scan indicates that her average Hz range of the Theta band is now 6.83hz, with most channels activating at 7.32hz. This suggests that most of her Theta range frequency is activating within a network, which can correlate with reduced neuroinflammation and improved cognitive abilities, such as supporting associative memory by facilitating the formation and retrieval of contextual and spatial associations [36,37]. According to Herweg, theta rhythms synchronize neuronal activity within the medial temporal lobe and between cortical areas, aiding in the encoding and recalling of episodic memories. “Scalp EEG studies may more reliably report increases in theta power associated with good memory because power measured at the scalp reflects large-scale neural synchrony, even as local theta amplitude is diminished.” These findings correlate with the patient's behavioral improvements of increased awareness, memory, attention, and cognition.

Overall Impact on Brain Function

The noteworthy effect sizes observed across these crucial electrode areas underscore the effectiveness of integrating neurofeedback and HBOT to foster brain recuperation after a severe TBI. Neurofeedback most likely boosted neural links and effectiveness by instructing the brain to manage its electrical activity autonomously, while HBOT supplied the essential oxygenation to facilitate neuronal restoration and mitigate inflammation. The combined impact of these treatments suggests that this led to a comprehensive enhancement in brain functionality, covering cognitive, motor, sensory, and emotional aspects.

Cognitive and communicative functioning and awareness (DRS) and (GOSE) 

From the pretest assessment conducted in 2021 to the posttest evaluation in 2024, with the DRS and the GOSE, the patient exhibited noteworthy progress across various cognitive and communicative functioning domains. Specifically, statistically significant improvements were observed in the patient's cognitive ability to engage in self-care activities such as feeding (p=0.046), toileting (p=0.046), and grooming (p=0.046) for each domain. 

In feeding, the patient showed a heightened cognitive awareness about her ability to feed herself and an enhanced capacity to communicate unambiguous information about when this activity should occur. Similarly, regarding toileting, the patient exhibited significant advancement in cognitive awareness of her toileting skills, alongside the ability to effectively convey information regarding the timing of this essential activity. Additionally, in grooming, the patient displayed notable improvement in her cognitive capacity to groom independently, coupled with the capability to articulate explicit information regarding the timing of grooming tasks. 

Furthermore, the patient's communication abilities were substantially enhanced, with a statistically significant increase (p=0.046) from the 2021 pretest to the 2024 post-test assessment. This improvement suggests a heightened level of self-awareness and environmental awareness. Notably, the patient demonstrated proficiency in providing accurate responses to inquiries regarding personal identity, spatial orientation, purpose, and temporal orientation, which was substantially enhanced, with a statistically significant increase in specificity. 

These findings underscore the patient's commendable progress in cognitive and communicative domains, reflecting a positive trajectory towards improved functional independence and overall quality of life. 

Functional Independence and Activities of Daily Living 

Despite cognitive gains, the patient’s functional independence remained compromised over the three years. In both 2021 and 2024, she necessitated round-the-clock nursing care and relied heavily on external assistance for activities of daily living (ADLs). While she demonstrated awareness of self-care tasks such as feeding, toileting, and grooming by 2024, she could not execute these activities independently, highlighting persistent functional deficits. 

The patient's dependence extended beyond the home environment, as reflected in her inability to shop or travel locally without assistance. These limitations represent a stark departure from her pre-injury autonomy and underscore the enduring impact of TBI on her instrumental activities of daily living (IADLs). 

*Psychosocial Functioning and Community Integration* 

The patient's challenged functional capacity influenced her psychosocial well-being and community integration. The GOSE assessment in 2024 revealed difficulties in social and leisure participation, with the patient engaging in activities less frequently than before her injury. Moreover, disruptions in familial and social relationships underscored the obstacles imposed by her TBI on interpersonal dynamics. 

The patient’s recent rise in psychological challenges is worth noting, as indicated by occasional disruptions in family and friendship interactions, implying a need for additional support for the patient's emotional well-being and coping strategies. It underscores the complex effects of TBI on psychological health outcomes. 

Employability and Vocational Functioning 

Consistent with her functional limitations, the patient’s employability status remained unchanged from 2021 to 2024. The enduring cognitive and physical impairments rendered her wholly unemployable, at present. This precludes her from assuming roles as a full-time worker, homemaker, or student. This restriction in vocational functioning underscores the pervasive and enduring nature of disability following TBI. 

Clinical implications and prognostic considerations 

This patient’s case underscores the complex interplay of cognitive, functional, and psychosocial domains in the aftermath of TBI. While her cognitive gains over three years are significant, persistent challenges in functional independence and community integration pose complications to her long-term rehabilitation and quality of life. 

The enduring psychosocial challenges and vocational hurdles underscore the imperative for comprehensive, multidisciplinary interventions targeting cognitive remediation, functional skill acquisition, and psychosocial support. Longitudinal follow-up and ongoing reassessment are essential to monitor the patient’s progress, address emerging needs, and optimize her rehabilitation trajectory. 

Limitations

This case report has its constraints. The study is based on a single case observation and employs only one neurofeedback and HBOT protocol. Therefore, to truly understand the therapeutic potential, it would be necessary to conduct additional analyses using various neurofeedback programs and hardware, as well as varying HBOT ATAs.

This study was primarily designed to test the feasibility of a straightforward and continuous regimen of neurofeedback and HBOT, using pretest and posttest qEEG and objective measures. This focus might limit the scope of the study. It may not provide comprehensive insights into the long-term effects or potential side effects of the neurofeedback and HBOT treatments. The study’s reliance on pretest and posttest measures might not capture the full range of patient responses over time. The study also assumes that the regimen can be implemented in a straightforward and ongoing manner, which might not be practical in all clinical settings or for all patients. While the study aims to highlight the overall utility of this approach for both diagnostic and therapeutic purposes, it may not fully explore other potential uses or benefits of the treatments. Therefore, further research is needed to address these limitations and provide a more comprehensive understanding of the treatments’ effects.

195 sessions might be considered excessive; however, specific lengthy neurofeedback protocols might require a high number of sessions to achieve significant and lasting changes in brainwave patterns and corresponding behavioral or cognitive improvements. With severe TBI, considering that neurofeedback is highly individualized, some participants might need more sessions to achieve the desired outcomes, especially if their initial qEEG showed significant dysregulation or if they have complex clinical presentations. Some patients respond more slowly to neurofeedback, necessitating a higher number of sessions for noticeable improvements. Also, multiple interim assessments were not administered, as pre-post framework existed as the overall assessment schedule.

Our findings show the potential effectiveness of a combined neurofeedback and HBOT treatment. The patient also received intensive physical therapy and other therapies. It is possible that different qEEG application patterns may be needed for different severe TBI patients. This could depend on several factors, including severity, duration, and most crucially, the unique pattern of treatments and qEEG changes observed in the initial recordings before the treatment begins.

To further our understanding, it’s crucial that researchers conduct more experimental studies. These studies should aim to evaluate the combined effects of neurofeedback and HBOT. By doing so, we can gain a more comprehensive understanding of their synergistic impact and potential benefits. This could pave the way for more effective treatment strategies in the future.

## Conclusions

The noteworthy effect sizes observed in this patient across key electrode regions may highlight the potential impact of combining neurofeedback and HBOT in promoting brain recovery following severe TBI. However, qEEG recordings and brain recovery are related aspects of neurological health but differ dimensionally. qEEG provides a detailed view of brain wave patterns, offering insights into cognitive states and potential abnormalities. Brain recovery, however, is the process of the brain healing and regaining functions after an injury or disorder, influenced by various factors like the patient’s health, injury severity, and treatment effectiveness. While qEEG contributes to understanding and monitoring brain recovery, it’s not the only indicator. Clinical assessments, patient-reported symptoms, and functional measures are also vital. Hence, qEEG and brain recovery should be considered when evaluating neurological health and treatment. Neurofeedback likely enhanced neural connectivity and efficiency by training the brain to self-regulate its electrical activity, while HBOT provided the necessary oxygenation to support neuronal repair and reduce inflammation. The potential combined impact of these therapies may have resulted in a more holistic improvement in brain function, encompassing cognitive, motor, sensory, and emotional domains. As reported by small, moderate, and high effect sizes, as well as DRS and GOSE results, this patient has made noteworthy gains in deep sleep, meditation, and early stages of sleep, relaxed, calm, and wakeful states, heightened alertness and intense focus, and higher cognitive functions like learning, memory, and information processing, along with communicative abilities. The patient's recovery process highlights the importance of an ongoing, holistic, patient-centered approach in her treatment planning. Recovery from severe TBI is a complex, long-term process involving physical healing and managing emotional, psychological, and social impacts. Healthcare professionals can create customized interventions through comprehensive assessment, including medical treatments, physiotherapy, psychological assistance, and social services. Continuous care and support are crucial, as recovery is not a singular event. This approach enables the patient to take charge of her recovery, overcome challenges, and work to regain meaningful participation in everyday life. This case serves as a powerful reminder of the resilience of the human spirit and the potential for healing with appropriate support and care.

## Appendices

**Table 4:** qEEG 19 Channel Scalp Electrode Placement Sites Dataset

Measure	Fp1AvPre	Fp2AvPre	F7AvPre	F3AvPre	FzAvPre	F4AvPre	F8AvPre	T7AvPre	C3AvPre	CzAvPre	C4AvPre	T8AvPre	P7AvPre	P3AvPre	PzAvPre	P4AvPre	P8AvPre	O1AvPre	O2AvPre		Fp1AvPost	Fp2AvPost	F7AvPost	F3AvPost	FzAvPost	F4AvPost	F8AvPost	T7AvPost	C3AvPost	CzAvPost	C4AvPost	T8AvPost	P7AvPost	P3AvPost	PzAvPost	P4AvPost	P8AvPost	O1AvPost	O2AvPost
Microvolts Squared (uV²)	1.93	0.80	1.76	2.06	1.65	1.99	0.79	0.78	1.54	0.9	1.17	0.7	3.00	4.31	2.97	1.94	3.93	4.72	4.61		0.58	0.37	0.6	0.87	0.94	0.78	0.84	1.30	0.51	1.13	0.38	1.83	1.05	0.74	0.96	1.15	2.21	2.39	2.50
Microvolts Squared (uV²)	0.89	0.48	0.90	1.20	1.10	1.09	0.43	0.47	1.01	0.83	0.77	0.48	1.59	2.26	1.60	1.02	2.31	2.76	2.98		0.46	0.35	0.56	0.70	0.76	0.59	0.72	1.14	0.60	1.28	0.48	1.55	1.17	0.83	1.02	0.93	2.51	2.16	2.49
Microvolts Squared (uV²)	2.88	2.13	1.93	3.25	4.13	4.36	2.83	1.17	2.82	3.65	2.75	2.25	6.94	6.70	5.77	2.75	11.86	23.18	20.34		4.35	5.19	5.13	4.56	5.22	5.10	6.53	4.71	3.83	8.25	3.32	10.94	9.46	7.16	8.01	6.12	38.08	39.13	49.28
Microvolts Squared (uV²)	2.19	0.87	1.50	1.56	1.42	1.29	0.88	0.58	1.52	2.15	0.97	0.91	1.94	1.97	1.36	1.19	3.33	5.63	4.81		0.62	0.61	0.76	0.96	0.92	1.06	0.95	0.93	0.80	2.19	0.69	1.65	1.71	1.14	1.17	1.28	5.33	5.12	6.06
Microvolts Squared (uV²)	4.41	1.25	2.90	2.02	1.33	1.21	1.02	0.65	1.46	1.45	0.98	0.79	1.91	1.5	1.10	1.02	2.64	3.9	3.14		0.57	0.41	0.60	1.13	0.83	1.09	0.88	0.74	0.71	1.54	0.65	1.24	1.16	0.79	0.94	0.94	2.37	2.42	2.94
Microvolts Squared (uV²)	2.11	0.56	1.58	1.01	0.46	0.41	0.46	0.26	0.59	0.40	0.32	0.31	0.88	0.52	0.35	0.33	1.06	1.51	0.89		0.19	0.11	0.20	0.44	0.3	0.44	0.38	0.25	0.20	0.37	0.18	0.48	0.36	0.20	0.25	0.23	0.57	0.53	0.67
Hertz (Hz)	1.46	1.46	1.46	1.46	1.46	1.46	1.46	1.46	1.46	1.46	1.46	1.46	1.46	1.46	1.46	1.46	1.46	1.46	1.46		1.46	1.46	2.20	1.46	1.46	1.46	1.46	2.2	1.46	1.46	1.46	1.46	1.46	1.46	1.46	1.46	1.46	1.46	1.46
Hertz (Hz)	3.91	3.91	3.91	3.91	3.91	3.91	3.91	4.15	4.15	4.39	3.91	3.91	3.91	3.91	3.91	3.91	3.91	3.91	3.91		7.32	7.32	7.32	4.15	4.15	7.32	7.32	7.32	7.32	7.32	4.39	7.32	7.32	7.32	7.32	7.32	7.32	7.32	7.32
Hertz (Hz)	10.74	10.74	10.74	10.01	10.74	10.74	10.74	10.25	10.01	10.01	10.50	10.50	10.50	10.74	10.74	10.74	10.01	10.50	10.01		10.50	10.50	10.50	10.50	5.22	10.50	10.50	10.25	9.28	9.28	10.25	9.28	9.52	10.50	10.50	10.50	9.28	10.50	10.25
Hertz (Hz)	19.78	13.92	19.78	13.92	13.92	13.92	13.92	13.92	13.92	14.65	13.92	13.92	13.92	13.92	13.92	13.92	13.92	13.92	13.92		13.92	13.92	13.92	13.92	13.92	13.92	13.92	13.92	13.92	13.92	13.92	13.92	13.92	13.92	14.16	13.92	13.92	13.92	13.92
Hertz (Hz)	21.97	20.75	19.78	20.75	20.75	20.26	20.02	19.78	19.78	19.78	19.78	19.78	20.02	19.78	19.78	19.78	19.78	19.78	20.02		19.78	20.51	20.51	19.78	19.78	19.78	20.75	20.26	23.44	19.78	21.24	20.51	21.00	19.78	20.51	20.51	19.78	19.78	19.78
Hertz (Hz)	30.52	30.27	30.27	30.76	29.79	30.27	30.52	30.76	30.52	29.79	29.79	29.79	29.79	29.79	29.79	30.27	29.79	30.52	30.52		30.76	30.76	30.03	29.79	30.27	30.27	30.03	30.03	30.27	30.52	30.52	31.01	30.52	30.27	30.27	29.79	30.27	30.27	30.76

**Table 5:** qEEG Frequency Band Parameters Outcomes – Pre (2021) - Post (2024) Dataset

Channel	2021DuV2	2021Dhz	2021TuV2	2021Thz	2021AuV2	2021Ahz	2021BuV2	2021Bhz	2021B2uV2	2021B2hz	2021GuV2	2021Ghz		2024DuV2	2024Dhz	2024TuV2	2024Thz	2024AuV2	2024Ahz	2024BuV2	2024Bhz	2024B2uV2	2024B2hz	2024GuV2	2024Ghz		DuV2DIFF	DhzDIFF	TuV2DIFF	ThzDIFF	AuV2DIFF	AhzDIFF	BuV2DIFF	BhzDIFF	B2uV2DIFF	B2hzDIFF	GuV2DIFF	GhzDIFF
Fp1-Av	1.93	1.46	0.89	3.91	2.88	10.74	2.19	19.78	4.41	21.97	2.11	30.52		0.58	1.46	0.46	7.32	4.35	10.5	0.62	13.92	0.57	19.78	0.19	30.76		1.35	0.00	0.43	-3.41	-1.47	0.24	1.57	5.86	3.84	2.19	1.92	-0.24
Fp2-Av	0.80	1.46	0.48	3.91	2.13	10.74	0.87	13.92	1.25	20.75	0.56	30.27		0.37	1.46	0.35	7.32	5.19	10.5	0.61	13.92	0.41	20.51	0.11	30.76		0.43	0.00	0.13	-3.41	-3.06	0.24	0.26	0.00	0.84	0.24	0.45	-0.49
F7-Av	1.76	1.46	0.90	3.91	1.93	10.74	1.50	19.78	2.90	19.78	1.58	30.27		0.60	2.20	0.56	7.32	5.13	10.5	0.76	13.92	0.60	20.51	0.20	30.03		1.16	-0.74	0.34	-3.41	-3.20	0.24	0.74	5.86	2.30	-0.73	1.38	0.24
F3-Av	2.06	1.46	1.20	3.91	3.25	10.01	1.56	13.92	2.02	20.75	1.01	30.76		0.87	1.46	0.70	4.15	4.56	10.5	0.96	13.92	1.13	19.78	0.44	29.79		1.19	0.00	0.50	-0.24	-1.31	-0.49	0.60	0.00	0.89	0.97	0.57	0.97
Fz-Av	1.65	1.46	1.10	3.91	4.13	10.74	1.42	13.92	1.33	20.75	0.46	29.79		0.94	1.46	0.76	4.15	5.22	10.5	0.92	13.92	0.83	19.78	0.30	30.27		0.71	0.00	0.34	-0.24	-1.09	0.24	0.50	0.00	0.50	0.97	0.16	-0.48
F4-Av	1.99	1.46	1.09	3.91	4.36	10.74	1.29	13.92	1.21	20.26	0.41	30.27		0.78	1.46	0.59	7.32	5.10	10.5	1.06	13.92	1.09	19.78	0.44	30.27		1.21	0.00	0.50	-3.41	-0.74	0.24	0.23	0.00	0.12	0.48	-0.03	0.00
F8-Av	0.79	1.46	0.43	3.91	2.83	10.74	0.88	13.92	1.02	20.02	0.46	30.52		0.84	1.46	0.72	7.32	6.53	10.5	0.95	13.92	0.88	20.75	0.38	30.03		-0.05	0.00	-0.29	-3.41	-3.7	0.24	-0.07	0.00	0.14	-0.73	0.08	0.49
T7-Av	0.78	1.46	0.47	4.15	1.17	10.25	0.58	13.92	0.65	19.78	0.26	30.76		1.30	2.20	1.14	7.32	4.71	10.25	0.93	13.92	0.74	20.26	0.25	30.03		-0.52	-0.74	-0.67	-3.17	-3.54	0.00	-0.35	0.00	-0.09	-0.48	0.01	0.73
C3-Av	1.54	1.46	1.01	4.15	2.82	10.01	1.52	13.92	1.46	19.78	0.59	30.52		0.51	1.46	0.6	7.32	3.83	9.28	0.8	13.92	0.71	23.44	0.2	30.27		1.03	0	0.41	-3.17	-1.01	0.73	0.72	0	0.75	-3.66	0.39	0.25
Cz-Av	0.90	1.46	0.83	4.39	3.65	10.01	2.15	14.65	1.45	19.78	0.40	29.79		1.13	1.46	1.28	7.32	8.25	9.28	2.19	13.92	1.54	19.78	0.37	30.52		-0.23	0.00	-0.45	-2.93	-4.6	0.73	-0.04	0.73	-0.09	0.00	0.03	-0.73
C4-Av	1.17	1.46	0.77	3.91	2.75	10.50	0.97	13.92	0.98	19.78	0.32	29.79		0.38	1.46	0.48	4.39	3.32	10.25	0.69	13.92	0.65	21.24	0.18	30.52		0.79	0.00	0.29	-0.48	-0.57	0.25	0.28	0.00	0.33	-1.46	0.14	-0.73
T8-Av	0.70	1.46	0.48	3.91	2.25	10.50	0.91	13.92	0.79	19.78	0.31	29.79		1.83	1.46	1.55	7.32	10.94	9.28	1.65	13.92	1.24	20.51	0.48	31.01		-1.13	0.00	-1.07	-3.41	-8.69	1.22	-0.74	0.00	-0.45	-0.73	-0.17	-1.22
P7-Av	3.00	1.46	1.59	3.91	6.94	10.50	1.94	13.92	1.91	20.02	0.88	29.79		1.05	1.46	1.17	7.32	9.46	9.52	1.71	13.92	1.16	21.00	0.36	30.52		1.95	0.00	0.42	-3.41	-2.52	0.98	0.23	0.00	0.75	-0.98	0.52	-0.73
P3-Av	4.31	1.46	2.26	3.91	6.70	10.74	1.97	13.92	1.50	19.78	0.52	29.79		0.74	1.46	0.83	7.32	7.16	10.5	1.14	13.92	0.79	19.78	0.20	30.27		3.57	0.00	1.43	-3.41	-0.46	0.24	0.83	0.00	0.71	0.00	0.32	-0.48
Pz-Av	2.97	1.46	1.60	3.91	5.77	10.74	1.36	13.92	1.10	19.78	0.35	29.79		0.96	1.46	1.02	7.32	8.01	10.5	1.17	14.16	0.94	20.51	0.25	30.27		2.01	0.00	0.58	-3.41	-2.24	0.24	0.19	-0.24	0.16	-0.73	0.10	-0.48
P4-Av	1.94	1.46	1.02	3.91	2.75	10.74	1.19	13.92	1.02	19.78	0.33	30.27		1.15	1.46	0.93	7.32	6.12	10.5	1.28	13.92	0.94	20.51	0.23	29.79		0.79	0.00	0.09	-3.41	-3.37	0.24	-0.09	0.00	0.08	-0.73	0.10	0.48
P8-Av	3.93	1.46	2.31	3.91	11.86	10.01	3.33	13.92	2.64	19.78	1.06	29.79		2.21	1.46	2.51	7.32	38.08	9.28	5.33	13.92	2.37	19.78	0.57	30.27		1.72	0.00	-0.20	-3.41	-26.22	0.73	-2.00	0.00	0.27	0.00	0.49	-0.48
O1-Av	4.72	1.46	2.76	3.91	23.18	10.5	5.63	13.92	3.90	19.78	1.51	30.52		2.39	1.46	2.16	7.32	39.13	10.5	5.12	13.92	2.42	19.78	0.53	30.27		2.33	0.00	0.60	-3.41	-15.95	0.00	0.51	0.00	1.48	0.00	0.98	0.25
O2-Av	4.61	1.46	2.98	3.91	20.34	10.01	4.81	13.92	3.14	20.02	0.89	30.52		2.50	1.46	2.49	7.32	49.28	10.25	6.06	13.92	2.94	19.78	0.67	30.76		2.11	0.00	0.49	-3.41	-28.94	-0.24	-1.25	0.00	0.20	0.24	0.22	-0.24
