# Correction: Longitudinal Outcomes of Neurofeedback and Hyperbaric Oxygen Therapy in Treating a Traumatic Brain Injury Patient: A Case Report 

**DOI:** 10.7759/cureus.c246

**Published:** 2025-08-13

**Authors:** Tami Peterson, JeAnnah Rose AbouAssaly, Wendy Bessler, Sheila Burgin, Robert Sherwin, Frederick Strale,

**Affiliations:** 1 Hyperbaric Oxygen Therapy, The Oxford Center, Brighton, USA; 2 Neurofeedback, The Oxford Center, Brighton, USA; 3 Neuropsychiatry, Advanced Neuropsychiatric Specialists, Farmington Hills, USA; 4 Hyperbaric Oxygen Therapy, Wayne State University School of Medicine, Detroit, USA; 5 Biostatistics, The Oxford Center, Brighton, USA

The Conflicts of Interest statement has been updated to declare the following:

Financial relationships: Tami Peterson, JeAnnah Rose AbouAssaly, Sheila Burgin, Robert Sherwin and Frederick Strale Jr. declare employment from The Oxford Center. Wendy Bessler declares employment from Advanced Neuropsychiatric Specialists.

